# A Dynamic Landscape for Antibody Binding Modulates Antibody-Mediated Neutralization of West Nile Virus

**DOI:** 10.1371/journal.ppat.1002111

**Published:** 2011-06-30

**Authors:** Kimberly A. Dowd, Christiane A. Jost, Anna P. Durbin, Stephen S. Whitehead, Theodore C. Pierson

**Affiliations:** 1 Viral Pathogenesis Section, Laboratory of Viral Diseases, National Institutes of Health, Bethesda, Maryland, United States of America; 2 Center for Immunization Research, Department of International Health, Johns Hopkins Bloomberg School of Public Health, Baltimore, Maryland, United States of America; 3 Laboratory of Infectious Diseases, National Institutes of Health, Bethesda, Maryland, United States of America; Institut Pasteur, France

## Abstract

Neutralizing antibodies are a significant component of the host's protective response against flavivirus infection. Neutralization of flaviviruses occurs when individual virions are engaged by antibodies with a stoichiometry that exceeds a required threshold. From this “multiple-hit” perspective, the neutralizing activity of antibodies is governed by the affinity with which it binds its epitope and the number of times this determinant is displayed on the surface of the virion. In this study, we investigated time-dependent changes in the fate of West Nile virus (WNV) decorated with antibody in solution. Experiments with the well-characterized neutralizing monoclonal antibody (MAb) E16 revealed a significant increase in neutralization activity over time that could not be explained by the kinetics of antibody binding, virion aggregation, or the action of complement. Additional kinetic experiments using the fusion-loop specific MAb E53, which has limited neutralizing activity because it recognizes a relatively inaccessible epitope on mature virions, identified a role of virus “breathing” in regulating neutralization activity. Remarkably, MAb E53 neutralized mature WNV in a time- and temperature-dependent manner. This phenomenon was confirmed in studies with a large panel of MAbs specific for epitopes in each domain of the WNV envelope protein, with sera from recipients of a live attenuated WNV vaccine, and in experiments with dengue virus. Given enough time, significant inhibition of infection was observed even for antibodies with very limited, or no neutralizing activity in standard neutralization assays. Together, our data suggests that the structural dynamics of flaviviruses impacts antibody-mediated neutralization via exposure of otherwise inaccessible epitopes, allowing for antibodies to dock on the virion with a stoichiometry sufficient for neutralization.

## Author Summary

Neutralizing antibodies are a critical aspect of protection from flavivirus infection. The primary targets of neutralizing antibodies are the envelope (E) proteins incorporated into virions. The neutralizing activity of antibodies is determined by the affinity with which they interact with the virion, and the total number of sites available for binding. In this study, we investigate the impact of dynamic motion of the viral E proteins on antibody-mediated neutralization. Using panels of monoclonal antibodies and immune sera, we demonstrate that the dynamic motion of virions significantly impacts antibody-mediated neutralization of West Nile and dengue viruses by modulating epitope accessibility. Increasing the length of the interactions between antibody and virus resulted in increased neutralization reflecting engagement of epitopes that are not exposed on the surface of the virion in its average state, but instead become accessible through the dynamic motion of E proteins. While examples of the impact of structural dynamics on antibody binding have been described previously, our data suggests this phenomenon plays a role in neutralization by all antibodies that bind the array of E proteins on the virion. Our data identifies epitope accessibility as a critical, yet dynamic, factor that governs the neutralizing activity of anti-flavivirus antibodies.

## Introduction

Flaviviruses are a group of ∼70 RNA viruses that cause morbidity and mortality on a global scale, with greater than 100 million human infections annually [1]. Viruses within this genus of medical concern include yellow fever virus, tick-borne encephalitis virus, Japanese encephalitis virus, dengue virus (DENV) and West Nile virus (WNV). WNV is a mosquito-borne flavivirus maintained in nature in an enzootic cycle with birds. WNV infections of humans result in a spectrum of clinical symptoms depending, in part, on the age and immune status of the individual. While most infections are sub-clinical, symptomatic cases range from self-limiting fever to acute flaccid paralysis and encephalitis [2]. Since its introduction into North America in 1999, as many as three million people have been infected by WNV [3], with ∼1000 severe infections occurring in the United States annually (www.cdc.gov). To date, there are no WNV-specific treatments or vaccines licensed for use in humans.

Flaviviruses are small spherical virions that encapsidate an ∼11 kb genomic RNA of positive-sense polarity [1]. This RNA is translated as a single polyprotein that is processed by viral and host cell proteases into ten functionally distinct proteins. Flaviviruses encode three structural proteins that comprise the virus particle and seven non-structural proteins that function to process the viral polyprotein, replicate the viral genome, and antagonize the host's protective response to infection [1], [4], [5], [6], [7], [8], [9], [10], [11]. Flaviviruses bud into the endoplasmic reticulum as immature viruses that incorporate 60 heterotrimeric spikes of the envelope (E) and precursor to membrane (prM) proteins [12], [13]. Maturation of virions during egress from the cell is associated with a pH-dependent change in the arrangement and oligomeric state of the E protein and cleavage of prM by a host cell furin-like serine protease [14], [15], [16]. While prM cleavage is a required step in the formation of an infectious virion [17], several lines of evidence suggest that a significant fraction of infectious virions retain some uncleaved prM [18], [19], [20], [21], [22]. The E protein is a class II fusion protein composed of three structurally distinct domains (domains I-III; E-DI-III) connected to the viral membrane by a helical stem region [23]. On mature virions, 180 copies of the E protein are arranged as anti-parallel dimers in an unusual herringbone T = 3 pseudo-icosahedral array [24], [25], [26]. In this configuration, E proteins are not quasi-equivalent, but exist in three distinct chemical environments defined by their proximity to the two-, three-, or five-fold symmetry axis. This arrangement adds complexity to the antigenic surface of virus particles as antibodies may not be able to bind all 180 E proteins due to steric constraints on antibody binding or occlusion of the epitope itself [27], [28].

Humoral immunity is a critical aspect of protection against flavivirus infection (reviewed in [29]). Eliciting a protective antibody response is a primary goal in the development of safe and effective flavivirus vaccines [30]. The majority of neutralizing antibodies against flaviviruses are directed against the E protein; neutralizing monoclonal antibodies (MAbs) have been identified that bind to all three E protein domains (reviewed in [29], [31]). While antibodies that bind the prM protein have been detected in humans, they possess limited neutralizing activity [32], [33], [34], [35], [36].

Antibody-mediated neutralization of WNV is a “multiple-hit” phenomenon that occurs when virions are engaged by antibody with a stoichiometry that exceeds a required threshold [29], [37]. Our estimate of this threshold is ∼30 antibody molecules per virion [38]. Two factors principally govern the neutralizing activity of an antibody. Antibody affinity (or avidity) controls the fraction of accessible epitopes on the intact virion occupied at a particular concentration of antibody. From a vaccination standpoint, eliciting antibodies that bind viral antigens with high affinity is desirable because they can reach the stoichiometric threshold required for neutralization at lower concentrations. However, high-affinity interactions do not always translate into significant functional potency. Because of steric constraints arising from the dense icosahedral arrangement of E proteins on the mature virion, not all epitopes are displayed on intact virions equivalently. Epitope accessibility is a second independent parameter that defines the circumstances that allow for neutralization, and differs markedly between antibodies that recognize structurally distinct epitopes [21], [28], [38], [39], [40]. In fact, many epitopes, including those recognized by antibodies commonly elicited *in vivo*, are not displayed on the surface of the mature virion enough times to allow for neutralization even when fully occupied [21], [38], [41].

An important concept of the “multiple-hit” model of neutralization is that infectious virions may be decorated with non-neutralizing quantities of antibody. Evidence in support of this includes increases in the neutralizing activity of antibodies observed in the presence of anti-IgG secondary antibodies or complement [37], [42], [43], and the phenomenon of antibody-dependent enhancement (ADE) of infection [44]. Because the rate of antibody-virion interactions is orders of magnitude faster than the rate of virus binding to cells, viruses are likely engaged by antibody for significant periods prior to productive interactions with target cells. Here, we investigated the fate of virions decorated by antibody with a stoichiometry that does not exceed the threshold required for neutralization. Our data revealed time- and temperature-dependent increases in neutralization of WNV that would not be predicted for static virions bound by antibodies under steady-state conditions. Because both viral proteins and intact virions are dynamic [45], [46], [47], [48], the increased neutralization can be explained by changes in epitope accessibility occurring as virions sample different states of a dynamic ensemble of conformations. That kinetic aspects of neutralization were observed for every WNV- and DENV-reactive MAb examined, as well as WNV-polyclonal immune sera, suggests a widespread impact of the dynamic motion of virions on epitope accessibility and antibody-mediated neutralization.

## Results

### Antibody modulates the infectious half-life of WNV in solution

**Figure 1:**
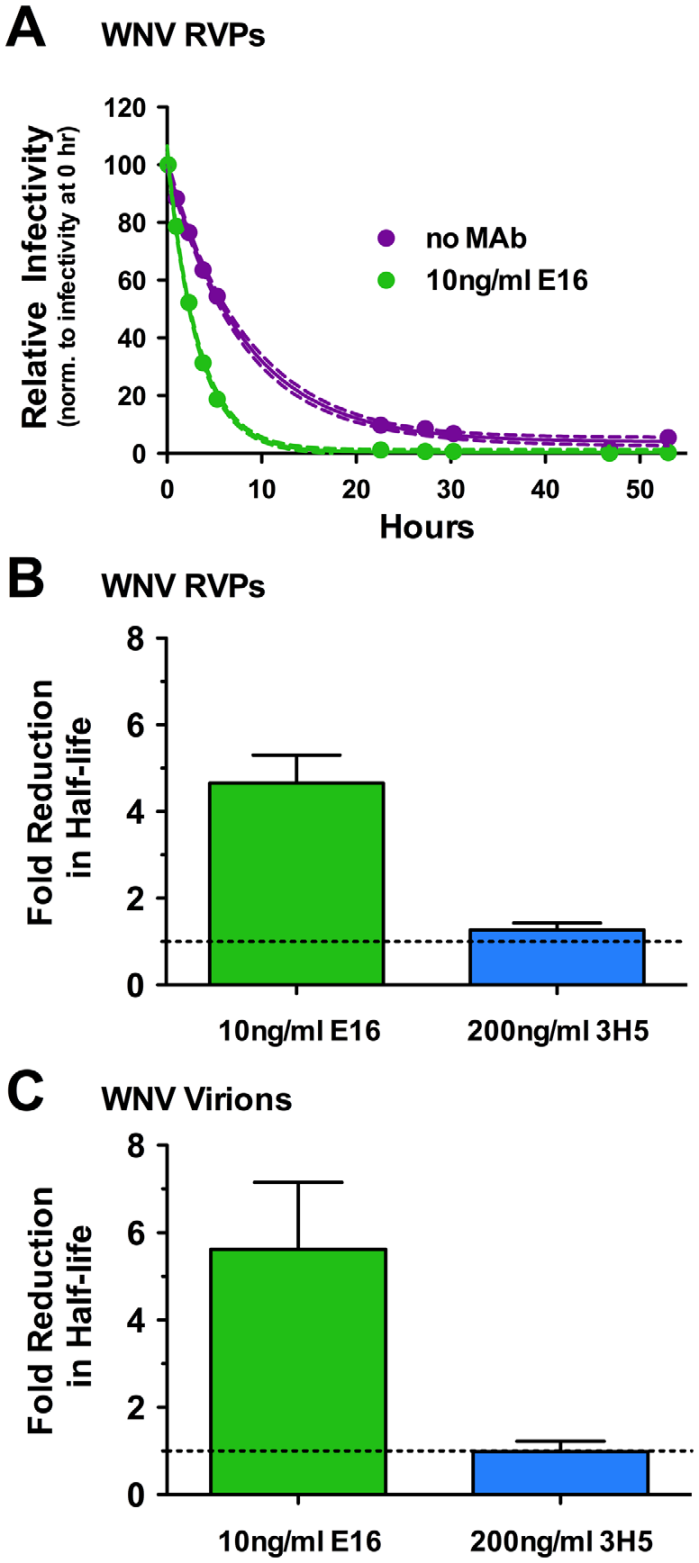
The functional half-life of WNV decreases in the presence of virus-specific antibody. (**A**) WNV RVPs were incubated in the absence or presence of sub-neutralizing concentrations (the EC_50_) of the MAb E16 for one hour at room temperature to allow binding to reach equilibrium. RVP-antibody complexes were then incubated at 37°C for the indicated times. The infectivity at each point was determined following infection of Raji-DC-SIGNR cells and monitored by flow cytometry 48 h post-infection. The data is presented normalized to levels obtained prior to incubation at 37°C (but after steady-state binding was reached) and fitted to a single-phase exponential decay to obtain the half-life. For the representative experiment shown, dotted lines represent 95% confidence intervals and error bars represent the standard error of duplicate measurements. The fold-reduction in the half-life of WNV RVPs (**B**) or infectious WNV (**C**) in the presence of sub-neutralizing quantities of MAb E16 (n = 23 and n = 2, respectively) or high concentrations of the DENV-specific MAb 3H5 (n = 2), as compared to the absence of antibody, are shown. Error bars represent the standard error.

**Figure 2:**
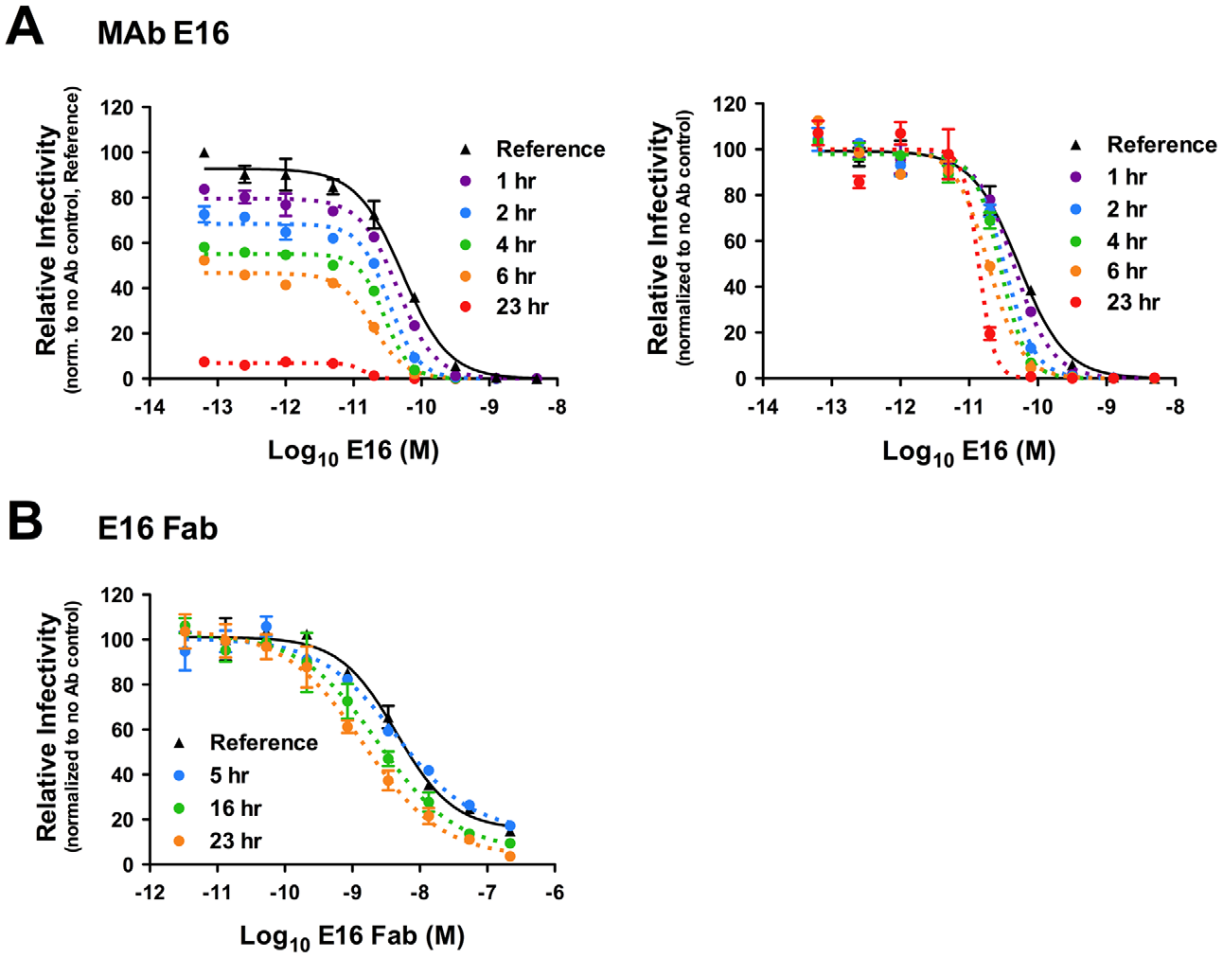
Kinetic changes in antibody-mediated neutralization of WNV infection by the DIII-specific MAb E16. Nine serial four-fold dilutions of MAb E16 (**A**) or E16 Fab fragments (**B**) were incubated with WNV RVPs for one hour at room temperature to allow binding to reach equilibrium. RVP-antibody complexes were then incubated at 37°C for incremental lengths of time before infecting Raji-DC-SIGNR cells. Infectivity was monitored by flow cytometry at 48 h post-infection. The reference curve represents RVP-antibody complexes added to target cells immediately after the room temperature incubation. For **A**, dose-response curves from a representative experiment are expressed relative to the infectivity of RVPs in the absence of antibody that were added to cells immediately after the room temperature incubation (**left panel**), or relative to the infectivity of RVPs in the absence of antibody at each individual time point (**right panel**). For **B**, the E16 Fab fragment results from a representative experiment are displayed relative to the infectivity of RVPs in the absence of antibody at each individual time point. Error bars display the standard error of duplicate infections. Results are representative of 19 and two independent experiments for A and B, respectively.

To investigate whether prolonged exposure of virions to “non-neutralizing” quantities of antibody has functional consequences, we performed a series of kinetic experiments with the WNV E-DIII-lateral ridge specific MAb E16 at a concentration sufficient to neutralize the infectivity of half the virus particles (the EC_50_). At this concentration, half the virions in a population are bound by antibody with a stoichiometry that exceeds the neutralization threshold, whereas the other half are bound by fewer antibody molecules. WNV reporter virus particles (RVPs) encoding a GFP reporter gene were incubated with 10 ng/ml E16 for one hour at room temperature to allow for steady-state binding (**Figure S1**). After the room temperature incubation, RVP-antibody complexes were shifted to 37°C for the indicated periods prior to the addition of target Raji-DC-SIGNR cells. The infectivity of RVPs in the presence and absence of antibody was determined by flow cytometric analysis, and is expressed relative to the level of infectivity observed when cells were added to viral immune complexes immediately after the initial room temperature incubation (**Figure 1a**). Using this approach, we observed an antibody-independent decrease in RVP infectivity with prolonged incubation at 37°C (intrinsic decay), consistent with our previously published findings [49]. Incubation in the presence of E16 resulted in a 4.7-fold reduction in the apparent half-life of WNV RVPs relative to the intrinsic rate of decay observed in the absence of antibody (n = 23; p<0.0001) (**Figure 1b**). A similar 5.6-fold change in the rate with which virions lost infectivity in the presence of E16 was observed using fully-infectious WNV (**Figure 1c**). Incubation of RVPs or virus with high concentrations of the DENV-specific MAb 3H5 did not impact WNV infectivity, indicating a requirement for virus-reactive antibody (**Figure 1b and c**). These results were not explained by increased binding of virions to tissue-culture plastic during prolonged incubation with antibody (p = .32, n = 5; **Figure S2a**), the action of complement (p = .17, n = 5; **Figure S2b**), or antibody-mediated aggregation (E16 Fab experiments discussed below; **Figure 2b**). Additionally, the rapid reduction in RVP infectivity in the presence of virus-specific antibody was still observed in the presence of a broad spectrum of protease inhibitors (p = .74, n = 2; **Figure S2c**), suggesting that the loss of infectivity cannot be explained by physical destruction of RVPs due to contaminating proteases.

### A kinetic aspect of neutralization by the WNV-specific MAb E16

We next evaluated the impact of kinetics on neutralization by E16 in a series of dose-response studies. WNV RVPs were incubated with serial four-fold dilutions of E16 at room temperature for one hour, followed by a shift to 37°C for the indicated times prior to the addition of target cells (**Figure 2a**). The dose-response profile of E16 obtained when cells were added immediately after the room temperature incubation required to achieve steady-state binding revealed the expected relatively steep sigmoidal curve (**Figure 2a**; reference curve) [38]. Dose-response curves obtained from samples incubated for varying lengths of time at 37°C prior to the addition of cells differed from the reference curve in several respects. First, the percentage of cells infected in the presence of low concentrations of antibody (the top of the sigmoidal dose-response curve) decreased as a function of increased incubation at 37°C, corresponding to the intrinsic decay of virus particles observed in the kinetic studies described above (**Figure 1**, **Figure 2a**; **left panel**). Second, increasing the length of incubation at 37°C shifted the EC_50_ to lower concentrations of antibody (**Figure 2a**; **right panel** (n = 19, p<0.0001)). Analysis of changes in infectivity over time revealed a 5.8-fold decrease in the apparent half-life at ∼10 ng/ml E16 relative to the antibody-independent intrinsic decay for RVPs alone (n = 18 independent dose-response experiments, p<0.0001), similar to the results obtained using the single antibody-concentration studies described in **Figure 1**. Time-dependent changes in dose-response curves were also observed with Fab fragments of E16 (∼3.8-fold decrease in EC_50_ after 24 hr incubation, n = 2) (**Figure 2b**), indicating the phenomenon does not reflect antibody-mediated virus aggregation.

### Kinetic aspects of the antibody dependent enhancement (ADE) profile of MAb E16

**Figure 3:**
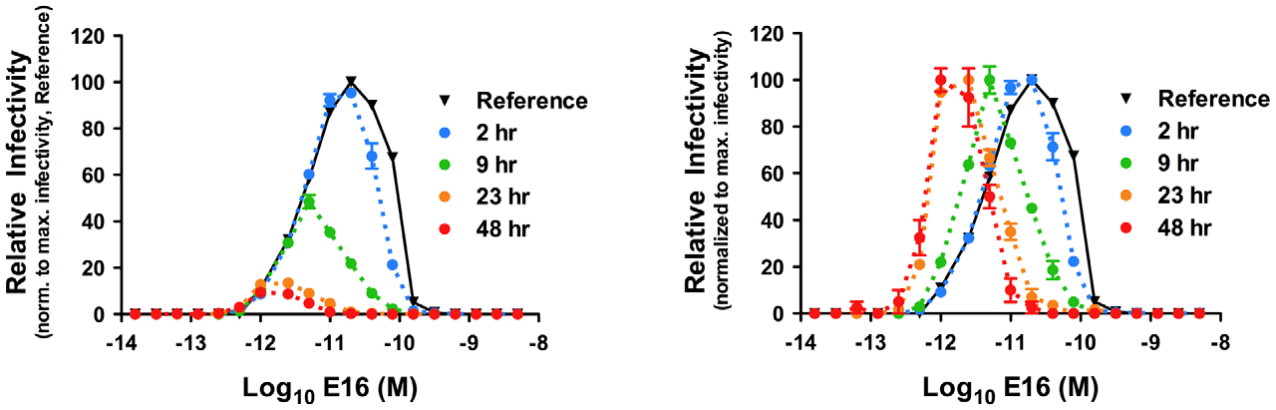
Kinetic changes in antibody dependent enhancement (ADE) of WNV infection by the MAb E16. Nine serial four-fold dilutions of MAb E16 were incubated with WNV RVPs for one hour at room temperature to allow binding to reach equilibrium. RVP-antibody complexes were then incubated at 37°C for incremental lengths of time before infecting K562 cells. Infectivity was monitored by flow cytometry at 48 h post-infection. The reference curve represents RVP-antibody complexes added to target cells immediately after the room temperature incubation. Dose-response curves from a representative experiment are expressed relative to the maximum infectivity of RVPs that were added to cells immediately after the room temperature incubation (**left panel**), or relative to the maximum infectivity of RVPs at each individual time point (**right panel**). Error bars display the standard error of duplicate infections. Results are representative of four independent experiments.

ADE describes the dramatic increase in infection of Fcγ-receptor-expressing cells in the presence of non- or weakly-neutralizing quantities of antibody and has been linked to severe clinical outcomes following secondary DENV infections of humans (reviewed in [50]). Antibody-mediated neutralization and ADE are two phenomena related by the number of antibodies bound to the virion [38], [51], therefore kinetic changes in the neutralization activity of an antibody should have a corresponding impact on the ADE profile as well. To explore this, kinetic dose-response curves were set up as described above, except using FcγIIb-expressing K652 target cells. Infection of K562 cells occurs through Fcγ-receptor-mediated uptake of antibody-virus immune complexes; in the absence of antibody, these cells are refractory to infection due to an inability to efficiently bind WNV. When immune complexes were added to cells immediately following the room temperature incubation required to achieve steady-state binding (**Figure 3**; reference curve), the expected bell shaped dose-response profile was observed. Incubation of antibody-virion complexes at 37°C prior to the addition of cells resulted in a marked change in the shape of the ADE profile. A reduction in the magnitude of ADE was observed over time, consistent with a decrease in the number of infectious virions in solution due to intrinsic decay (**Figure 3**; **left panel**). Notably, prolonged incubation resulted in a reduction in the concentration of E16 at which maximal ADE was observed (**Figure 3**; **right panel**), corresponding to the reduction in the EC_50_ observed above (**Figure 2a**; **right panel**).

Because the kinetic neutralization and enhancement experiments presented above were designed to allow for steady-state binding of the virion by antibody prior to incubation at 37°C (validated in **Figure S1**), changes in virus infectivity over time should not reflect continued engagement of individual virions by increasing numbers of antibody molecules. However, an important assumption of this model is that the number of epitopes on the virion does not change.

### Dynamic motion changes epitope accessibility in a time- and temperature-dependent manner

To explore the role of dynamic motion of the virion in WNV neutralization, we took advantage of the fact that the neutralizing activity of several classes of antibodies is significantly limited by epitope accessibility [21], [39], [40]. MAb E53 is a high affinity DII-fusion loop-reactive antibody that has a limited capacity to neutralize mature virions because its epitope is buried on the surface of the virus particle. Even in the presence of saturating concentrations of antibody, E53 does not bind mature virions enough times to exceed the stoichiometric requirements for neutralization [21]. In support, structural studies revealed that E53 Fab fragments were only capable of binding E proteins of the heterotrimeric spikes present on immature virions [52]. For E53 to engage a mature virion with a stoichiometry that exceeds the neutralization threshold, changes in the accessibility of its otherwise cryptic epitopes would be required.

**Figure 4:**
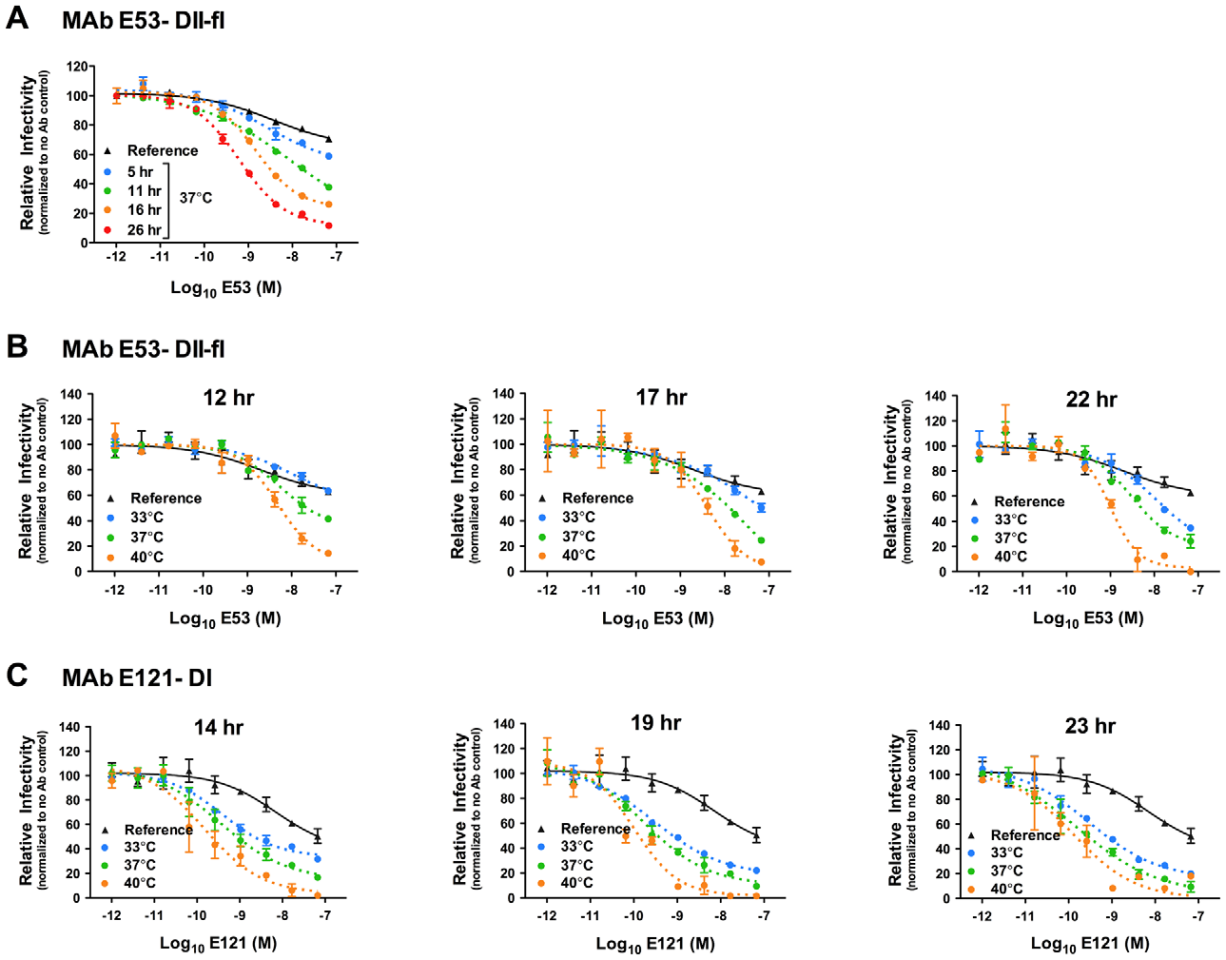
Time- and temperature-dependent increases in neutralization by antibodies that bind cryptic determinants on the mature virion. Nine serial four-fold dilutions of the DII-fusion loop (fl)-reactive MAb E53 (**A** and **B**) or the DI-reactive MAb E121 (**C**) were incubated with mature WNV RVPs for one hour at room temperature to allow binding to reach equilibrium. RVP-antibody complexes were then incubated at 37°C only (**A**) or at 33°C, 37°C, and 40°C (**B** and **C**) for incremental lengths of time before infecting Raji-DC-SIGNR cells. Infectivity was carried out at 37°C and monitored by flow cytometry at 48 h post-infection. The reference curve represents RVP-antibody complexes added to Raji-DC-SIGNR cells immediately after the room temperature incubation. Dose-response curves from representative experiments are expressed relative to the infectivity of RVPs in the absence of antibody at each individual time point. Error bars display the standard error of duplicate infections. Results are representative of five and three independent experiments for MAb E53 and E121, respectively.

In agreement with our previous findings [21], MAb E53 failed to significantly neutralize a homogeneous population of mature WNV RVPs when antibody-virion complexes were added to cells following a one hour incubation at room temperature (**Figure 4a and b**, reference curves). Incubation at 37°C for increasing time intervals prior to infection revealed a gradual increase in antibody potency, with more than half the virions incubated at 37°C for 26 hours becoming susceptible to neutralization (**Figure 4a**). Because the E53 epitope is not accessible on the highly ordered surface of the mature virion [52], the increase in potency requires changes in exposure of the fusion loop epitope over time as the E proteins on the surface of the virion sample different conformations at equilibrium. Binding of E53 has the potential to stabilize the epitope in an exposed conformation that occurs only transiently in the absence of antibody. That significant neutralization requires considerable time at 37°C likely reflects the relatively large number of epitopes that must become accessible on the average virion in order to exceed the neutralization threshold (**Figure S3**). If increased neutralization by E53 reflects dynamic movement of the virion, one would predict it would be both time- and temperature-dependent. To test this, we performed additional experiments with longer incubation times and a range of temperatures. Increasing the temperature to 40°C enhanced neutralization as compared to 37°C (**Figure 4b**, compare green to orange curves), whereas decreasing the temperature to 33°C reduced neutralization capacity (**Figure 4b**, compare green to blue curves). Similar results were observed for the E-DI specific MAb E121 shown previously to bind an epitope that is poorly accessible on the mature virion (**Figure 4c**) [21], [39].

### Viral dynamics broadly impact antibody-mediated neutralization of flaviviruses

**Figure 5:**
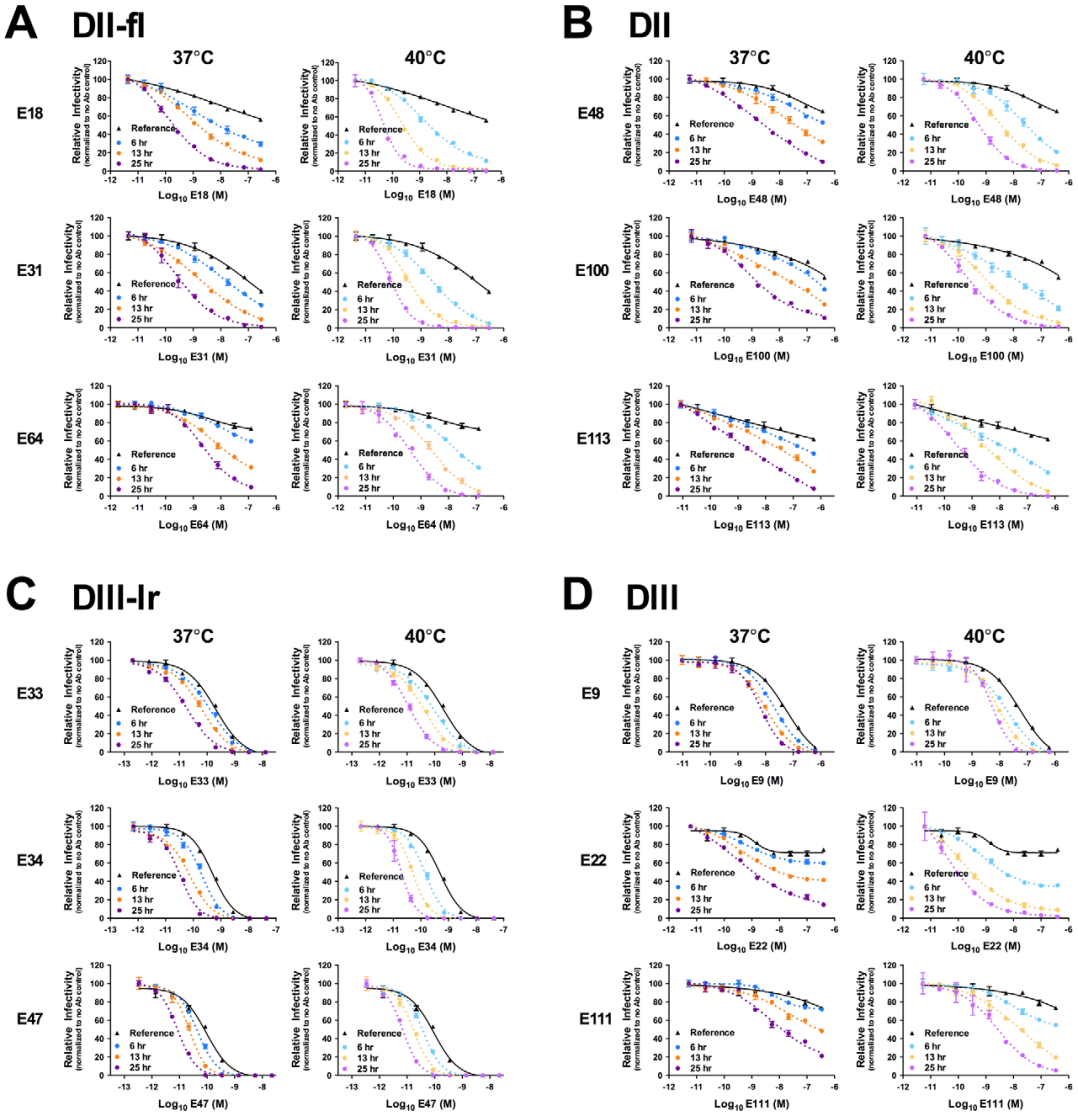
Kinetic changes in neutralization occur for WNV MAbs specific for structurally distinct epitopes. Nine serial four-fold dilutions of various WNV MAbs were incubated with mature WNV RVPs for one hour at room temperature to allow binding to reach equilibrium. RVP-antibody complexes were then incubated at 37°C or 40°C for incremental lengths of time before infecting Raji-DC-SIGNR cells. Infectivity was monitored by flow cytometry at 48 h post-infection. The reference curve represents RVP-antibody complexes added to Raji-DC-SIGNR cells immediately after the room temperature incubation. Dose-response curves are expressed relative to the infectivity of RVPs in the absence of antibody at each time point. Error bars display the standard error of duplicate infections. Results are representative of two independent experiments. MAbs selected for study were specific for epitopes on the DII-fusion loop (DII-fl) (**A**), the DII-central interface, DII-dimer interface, and DII-hinge interface (E48, E100, and E113, respectively) (**B**), the DIII-lateral ridge (DIII-lr) (**C**), and epitopes within DIII that fall outside of the lateral ridge (**D**).

**Figure 6:**
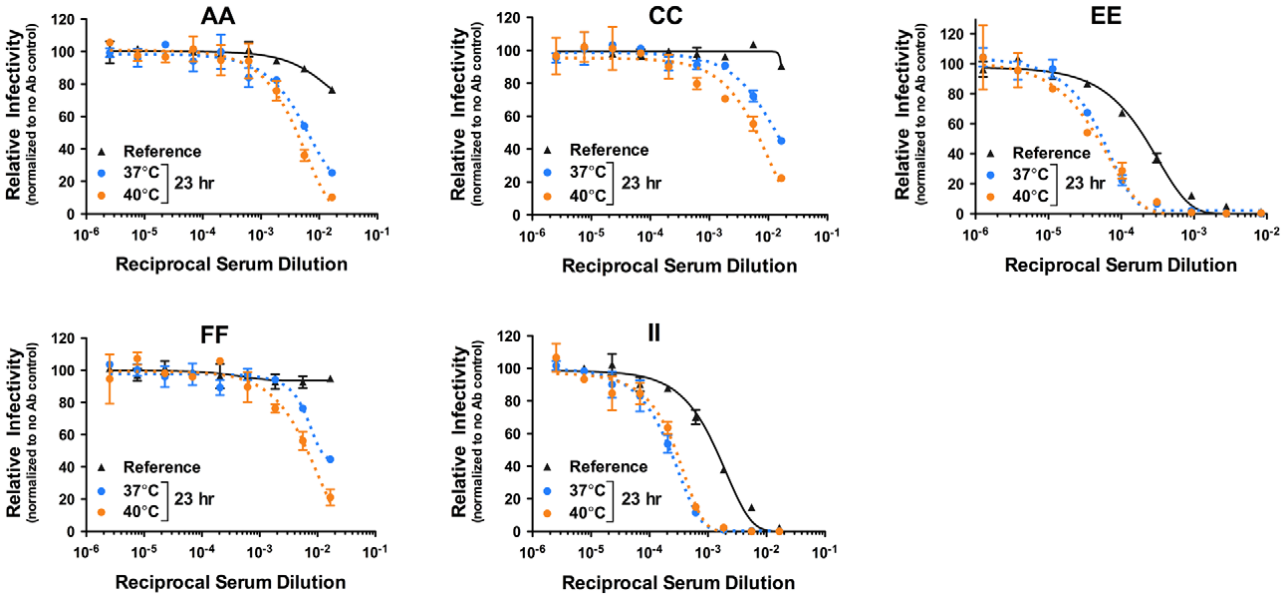
Kinetic effects of WNV neutralization by polyclonal sera. Nine serial three-fold dilutions of five heat-inactivated polyclonal WNV immune sera samples were incubated with mature WNV RVPs for one hour at room temperature to allow binding to reach equilibrium. RVP-antibody complexes were then incubated at 37°C or 40°C for incremental lengths of time before infecting Raji-DC-SIGNR cells. Infectivity was carried out at 37°C and monitored by flow cytometry at 48 h post-infection. The reference curve represents RVP-antibody complexes added to Raji-DC-SIGNR cells immediately after the room temperature incubation. Dose-response curves from a representative experiment are expressed relative to the infectivity in the absence of antibody at each individual time point. Error bars display the standard error of duplicate infections. Data is representative of two independent experiments.

To investigate whether kinetic effects on antibody-mediated neutralization are a common phenomenon, we performed additional studies using a panel of 12 WNV MAbs. In each case, we observed time- and temperature-dependent increases in neutralization, similar to our results with E16, E53, and E121 (**Figure 5**). Collectively, the 15 WNV MAbs used in the current study bind epitopes distributed across all three domains of the E protein and are characterized by varying degrees of functional potency [39], [53]. We next performed experiments using previously characterized immune sera from five recipients of a phase I clinical trial of a live attenuated WNV vaccine [21] (A. Durbin, S. Whitehead, and colleagues, unpublished data). After a one hour room temperature incubation to allow for steady-state binding, virus-sera mixtures either were used to immediately infect cells (**Figure 6**, reference curves) or incubated an additional 23 hours at 37°C or 40°C before infection (**Figure 6**, blue and orange curves, respectively). We found that the polyclonal mixtures of antibody present in immune sera behaved in a manner similar to MAb of defined specificity.

**Figure 7:**
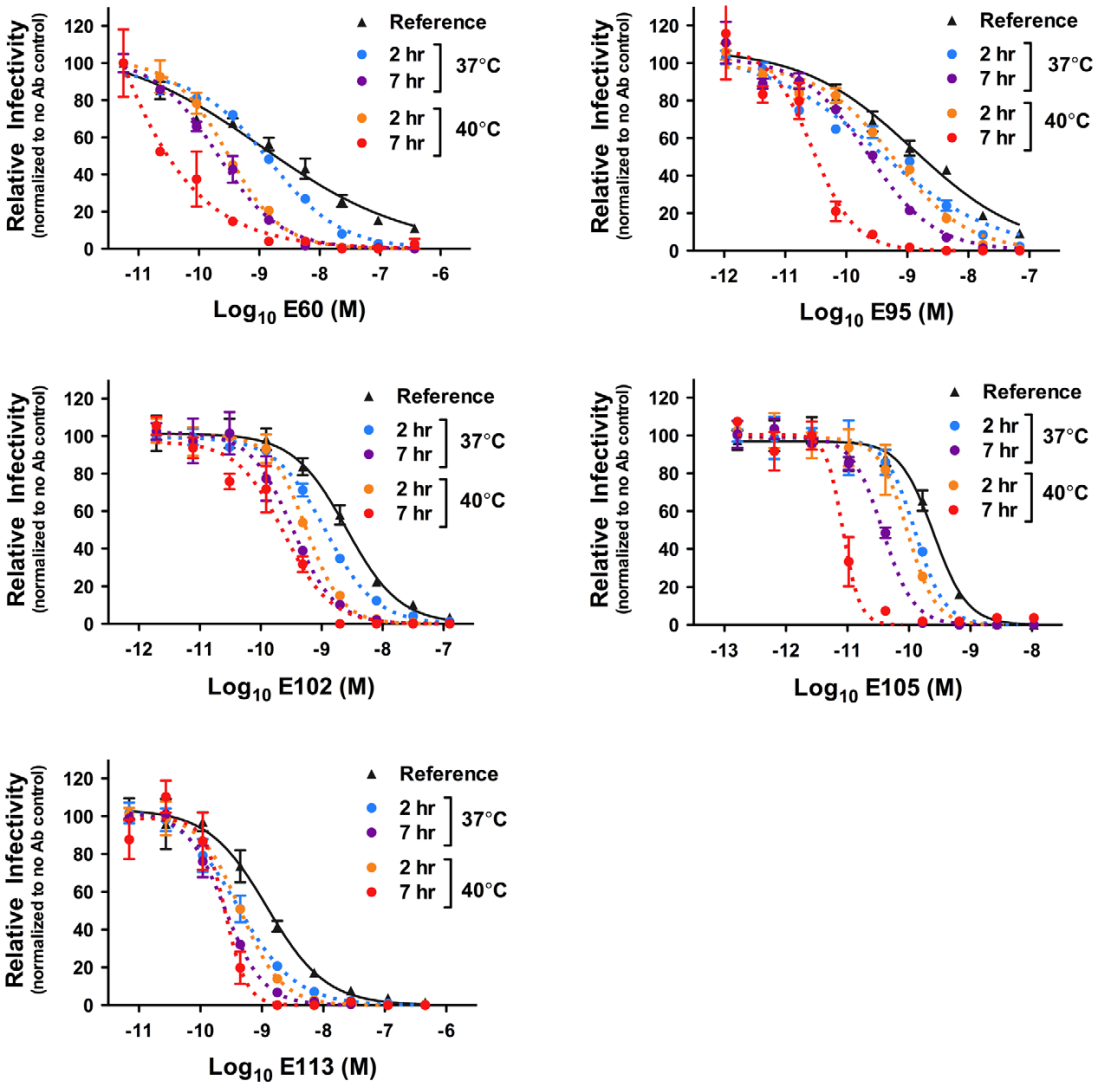
A kinetic aspect of neutralization is also observed with DENV. Nine serial four-fold dilutions of various DENV-1-reactive MAbs were incubated with DENV-1 RVPs for one hour at 37°C to allow binding to reach equilibrium. RVP-antibody complexes were then incubated at 37°C or 40°C for incremental lengths of time before infecting Raji-DC-SIGNR cells. Infectivity was carried out at 37°C and monitored by flow cytometry at 48 h post-infection. The reference curve represents RVP-antibody complexes added to Raji-DC-SIGNR cells immediately after the one hour incubation at 37°C. Dose-response curves from a representative experiment are expressed relative to the infectivity in the absence of antibody at each individual time point. Error bars display the standard error of duplicate infections. Data is representative of three (E95, E105) or two (E60, E102, E113) independent experiments. E95, E102, E105, and E113 are DIII-reactive MAbs specific for the G-strand, BC-loop, BC/DE/FG loops, and A-strand, respectively. E60 is a WNV-specific MAb that is cross-reactive for the DENV DII-fusion loop.

To extend our observations to another flavivirus, we performed analogous studies with DENV serotype 1 (DENV-1) RVPs and a panel of previously characterized DENV-1 E protein-reactive MAbs [53], [54]. After incubating RVPs and antibody at 37°C for one hour to allow steady-state binding to occur, RVP-antibody complexes were either immediately used to infect cells (**Figure 7**, reference curves) or further incubated for two or seven additional hours at 37°C and 40°C before infection (**Figure 7**, dotted curves). Because it was established previously that some antibodies do not bind DENV efficiently at low temperatures [46], incubation at 37°C was used to establish steady-state binding (**Figure S1**). As observed in our studies with WNV, time- and temperature-dependent increases in neutralization potency were observed (**Figure 7**). Interestingly, shorter incubation times were necessary as compared to experiments with WNV, due to a more rapid intrinsic decay rate of DENV as compared to WNV, which has previously been reported [49]. This was also demonstrated in parallel studies of WNV and DENV-1 RVPs with the cross-reactive MAb E60; significant increases in neutralization were observed more rapidly with DENV than WNV (**Figure S4**). Altogether, these results suggest that DENV exists in a more dynamic state than WNV.

## Discussion

The existence of high-resolution structures of the E proteins of flaviviruses, and their arrangement on the surface of the virion at different stages of the viral lifecycle, have been a powerful tool for studying how these viruses interact with the host (reviewed in [6], [26]). Interpretation of these structures in the context of biological systems is complicated by the fact that they represent the average state of what is likely a very dynamic ensemble of conformations sampled by virions at equilibrium. The structural dynamics of non-enveloped viruses have been studied extensively (reviewed in [45], [55]). Limited proteolysis studies of the Flock house virus strongly suggest the capsid proteins “breathe”, allowing proteases access to internal structures predicted to be inaccessible on an intact and non-dynamic virion [47]. Furthermore, drugs that prevent the dynamic movement of the picornavirus human rhinovirus 14 have been shown to effectively inhibit infectivity [48]. While the dynamics of enveloped viruses have been studied less extensively, examples of temperature- and time-dependent antibody reactivity have been described for several viruses [46], [56], [57]. The DENV group-reactive neutralizing MAb 1A1D-2 binds an epitope on the A-strand of E-DIII that is poorly accessible on proteins proximal to each of the three symmetry axes of the mature virion. Binding of this MAb is temperature-dependent (does not occur at 4°C) and appears to trap E proteins on the virion surface in a conformation distinct from the herringbone icosahedral arrangement of the mature virion [46].

Overall, prior studies implicating a kinetic component of antibody-mediated neutralization have focused on either a single MAb (DENV MAb 1A1D-2, influenza MAb Y8-10C2) or a panel of MAbs that recognizes a similar epitope (MAbs specific for the membrane-proximal external region (MPER) of HIV gp41), with the implication that such antibodies were atypical in their ability to bind buried or inaccessible epitopes [57], [58], [59]. In this study we provide functional evidence identifying the widespread impact of the dynamic movement of flaviviruses on neutralization by all antibodies that bind the E protein; a kinetic aspect of neutralization appears to be the rule rather than the exception.

Our data suggest that the structural dynamics of virions has the potential to modulate the potency of all antibodies that bind E proteins arrayed on the surface of the virion through changes in epitope accessibility. This is illustrated most dramatically by antibodies that bind poorly accessible determinants on the mature virion, such as the WNV DII-fusion loop-reactive MAb E53. Neutralization of mature WNV by E53 is restricted by the number of times the antibody can bind the virion. E53 binds the E protein only when associated with prM as heterotrimeric spikes that project off the surface of the immature virus particle [21], [52]. While homogeneous populations of mature WNV are not efficiently neutralized by E53 when assayed using conventional approaches, increasing the time the virus is incubated with antibody resulted in a marked increase in neutralization activity (>100-fold) (**Figure 4a and b**). Because the virus cannot revert to an immature configuration once prM cleavage occurs and the virion is released from the cell [15], this dramatic increase in neutralization can only be explained by exposure of the DII-fusion loop epitope through dynamic motion of viral E proteins.

The neutralization activity of every monoclonal (n = 20) and polyclonal (n = 5) antibody assayed in this study was enhanced by increasing the time the virion was exposed to antibody prior to the addition of target cells, including antibodies previously demonstrated to be non- or weakly-neutralizing using standard assays. This reflects the fact that as the number of accessible epitopes on the individual virion increases as a consequence of dynamic motion, the fraction of them that must be bound in order to exceed the stoichiometric threshold (percent occupancy) is reduced; neutralization can then occur at lower concentrations of antibody. However, the magnitude of this kinetic effect was not uniform among antibodies localizing to different epitopes. This may reflect differences in the number of times an antibody binds the average state of the virion relative to the threshold number of antibodies required for neutralization, as well as the rate at which additional epitopes are made available for binding (**Figure S3**). In contrast to the cryptic nature of the DII-fusion loop epitope recognized by E53, the potently neutralizing MAb E16 is specific for a relatively accessible determinant displayed on the lateral ridge of DIII [53]. Cryo-electron microscopy studies indicate this antibody binds 120 out of 180 E proteins incorporated into mature virions; the remaining 60 E proteins proximal to the five-fold symmetry axis of the virion cannot be bound due to steric conflicts among the tightly clustered DIII epitopes [27], [28]. An increase in the accessibility of these additional epitopes on the virion through dynamic motion would translate into a modest reduction in the occupancy requirements for neutralization by E16, in agreement with the 4.0-fold increase in antibody potency (n = 11, range 2.3–6.8) observed in our studies after ∼24 hours incubation. A similar 3.8-fold increase after ∼24 hours was observed when E16 Fab fragments were used (n = 2, range 3.2–4.5).

The dynamic motion of virions has the potential to increase antibody potency by providing access to otherwise cryptic antibody-binding determinants. Of note, mapping studies suggest that many epitopes on the mature virion are poorly accessible for antibody binding [38], [39], [40], [46]. Antibodies do not induce viral breathing, but rather stabilize conformations of the E protein that exist as part of the ensemble of conformations sampled by the virion at equilibrium. The longer the virion remains exposed to antibody, the more opportunities exist for engagement of an otherwise inaccessible epitope, allowing for time-dependent increases in the stoichiometry with which antibodies decorate virions. If changes in epitope accessibility are the underlying mechanism of the kinetic aspects of neutralization, there should be a limit to the increase in potency observed over time. Eventually, dynamic virion structures should expose all potential epitopes, and these will become fixed in place by antibody binding, yielding a neutralization profile determined by the relationship between antibody occupancy and the stoichiometric threshold. In support of this, increases in neutralization and changes in the ADE curves for E16 no longer occurred when incubations longer than 24 hours were performed (**Figure S5**).

In addition to exposing more epitopes for antibody binding, time-dependent changes in the antigenic surface of the virus particle may also allow engagement of the virion with increased affinity, via bivalent interactions among E proteins in conformations not present in the average state, as well as cooperative effects during antibody binding. That the kinetic impact on neutralization by E16 was observed using both intact antibodies and Fab fragments incapable of cross-linking virions indicates that increases in antibody potency do not reflect antibody-mediated aggregation among virions. Importantly, all of the experiments included in our study were performed using conditions of antibody excess, and yielded results that were independent of the concentration of virus in the assay. In contrast, the aggregation of antigens by antibodies is dependent on the antibody-antigen ratio.

Our results suggest that changes in the antibody-mediated neutralization of DENV occur more rapidly than with WNV. One interpretation of this result is that DENV virions are more dynamic than those of WNV, allowing more rapid access to otherwise inaccessible determinants. In the absence of antibody, preparations of DENV become less infectious at a faster rate than observed with WNV, consistent with prior studies [49]. Additionally, kinetic changes in neutralization with the cross-reactive MAb E60 occur at a faster rate with DENV-1 than WNV RVPs when compared in parallel studies (**Figure S4**). While we do not yet understand, in molecular terms, why viruses lose infectivity over time, one possibility is that the intrinsic decay of flaviviruses is a consequence of structural dynamics. Viruses sampling multiple conformations in dynamic equilibrium may not always return to the average state because moving backwards may no longer be the most energetically favorable path. Additional evidence of time-dependent structural changes to the virus population is demonstrated by differences in the intrinsic decay rate of WNV observed when different cell types are used to measure infectivity. The rate of decay of viruses was ∼2.7-fold more rapid when assayed on Raji-DC-SIGNR cells as compared to a K562 cell line expressing the same attachment protein (n = 6, p<0.0001). Thus, the observed intrinsic decay cannot be attributed solely to the physical destruction of the virus, and suggests the additional possibility that not all conformations in a heterogeneous ensemble of virions are equally infectious on different cell types. Of interest, E proteins on individual virions in conformations that may no longer contribute functionally to fusion may also stably expose a different array of epitopes.

Our data suggest that the circumstances of antibody-virion interactions may significantly impact the fate of the virion immune complex. Standard *in vitro* neutralization assays for flaviviruses generally include a short pre-incubation (∼1 hr) of antibody and virus prior to infection of target cells; this incubation presumably allows the binding reaction between antibody and cognate epitope to reach steady-state. However, depending on the extent to which a virion is structurally dynamic (which controls the rate at which epitopes may become transiently accessible), the target cell type, and the volume of infection *in vitro*, this presumption may be inaccurate. Because increases in the neutralization activity of DENV-reactive antibodies that bind dynamically exposed epitopes occur rapidly (within two hours) (**Figure 7**), the interaction of DENV with antibodies may never truly reach steady-state. From this perspective, the length of time antibody is incubated with DENV is a variable that cannot be ignored. While antibody-mediated neutralization activity measured *in vitro* using standard plaque reduction neutralization tests (PRNT) generally correlates with protection *in vivo* [39], [53], [54], [60], [61], [62], this is an imperfect relationship. Antibodies with limited neutralization activity have been shown to protect in animal models of flavivirus infection [39], [54], [62]. While this may reflect the direct contributions of effector functions of antibodies *in vivo*, it is also possible that existing assays of the functional properties of antibodies have limitations. Considering the contribution of the structural dynamics of the virion when designing neutralization studies merits a systematic evaluation.

The impact of the dynamic exposure of viral epitopes *in vivo* remains uncertain. Virtually nothing is known about the relevant concentrations and volumes that govern antibody-virion interactions in the tissues where many of the key events in the pathogenesis of these viruses occur. Kinetic changes in neutralization occur gradually over time as dynamic motion provides new opportunities for engagement of virions with a stoichiometry sufficient for neutralization (**Figure S3**). Thus the rate of virus entry *in vivo* is also an important, yet unknown, parameter that defines the extent to which this phenomenon will contribute to protection of the host. Of interest, the kinetics of WNV binding to target cells *in vitro* occurred rather slowly (with maximal binding requiring ∼3 hours) even in the presence of the high affinity attachment factor DC-SIGNR (**Figure S6**). As DENV appears to be extremely dynamic, with kinetic effects on neutralization observed almost immediately, the impact of viral breathing on neutralization *in vivo* cannot be discounted. Because the kinetics of neutralization are increased by an elevated temperature, it is interesting to speculate that certain classes of antibodies, such as those recognizing the fusion loop epitope commonly observed in infected individuals, may function better than previously anticipated in the context of the febrile response. Resolving this question awaits the development of approaches to quantitatively and directly measure antibody-mediated neutralization *in vivo*.

Neither proteins, nor intact virions, are static structures. Our findings are consistent with a model in which the dynamic motion of flaviviruses provides an opportunity for antibodies to engage virions at otherwise inaccessible epitopes to reach a stoichiometry sufficient for neutralization. Given time, all of the E protein-reactive antibodies investigated were able to block virus infection, even those described originally as non-neutralizing using conventional assays [39]. These results add to the complexity of our understanding of the functional properties of antibodies and suggest new avenues of investigation and analysis into the widespread and unappreciated impact of the dynamic motion of virions as moving targets for antibody recognition.

## Materials and Methods

### Cell lines

Cell lines were maintained at 37°C in the presence of 7% CO_2_. HEK-293T cells were grown in complete Dulbecco's modified Eagle medium (DMEM) containing Glutamax (Invitrogen, Carlsbad, CA) and supplemented with 10% fetal bovine serum (FBS; (HyClone, Logan, UT)) and 100 U/ml penicillin-streptomycin (PS; (Invitrogen, Carlsbad, CA)). K562 and Raji cell lines were maintained in RPMI-1640 medium containing Glutamax (Invitrogen, Carlsbad, CA) and supplemented with 10% FBS and 100 U/ml PS.

### WNV and DENV immune sera

Neutralization studies were performed using sera obtained from recipients of a candidate WNV vaccine. Sera from five participants of a Phase I double-blinded, placebo-controlled study designed to evaluate the safety and immunogenicity of a single dose of live attenuated WNV/DENV-4 vaccine were obtained and characterized previously [21]. The clinical study was conducted at the Center for Immunization at the Johns Hopkins Bloomberg School of Public Health under an investigational new drug application reviewed by the United States Food and Drug Administration.

### Production of WNV and DENV RVPs

Reporter virus particles (RVPs) are pseudo-infectious virions produced by genetic complementation of a WNV sub-genomic replicon with the structural genes *in trans*. Flavivirus replicons do not encode intact genes for the three structural proteins of the virus, thus RVPs that encapsidate these sub-genomic RNAs are capable of only a single round of infection. WNV and DENV RVPs have been used extensively to characterize the functional properties of anti-flavivirus antibodies [21], [38], [39], [42], [63], [64].

RVPs were produced by transfection of HEK-293T cells with DNA plasmids encoding the structural genes and a sub-genomic WNV replicon as described previously [38], [65]. Standard preparations of WNV RVPs were produced using plasmids encoding a GFP-expressing replicon, WNV C-prM-E, and pcDNA3.1 with a 1∶3∶0.5 ratio by mass. Because standard preparations of WNV RVPs retain detectable amounts of uncleaved prM, homogeneous populations of mature WNV RVPs were produced using the plasmids above except that a plasmid encoding human furin was used in place of pcDNA3.1 to promote efficient cleavage of prM [21]. Over-expression of furin in this context has been shown to reduce the amount of uncleaved prM protein in populations of RVPs to levels that are no longer detectable by Western blot [21], [66]. Standard RVPs composed of the structural genes of the DENV Western Pacific strain (serotype I: genotype IV) were produced as described above by substituting a plasmid encoding DENV structural genes in place of the WNV construct [49]. All transfections were performed using Lipofectamine LTX (Invitrogen, Carlsbad, CA) in accordance with the manufacturer's instructions. RVPs were harvested at 48 h post-transfection, filtered through a 0.22 µM filter, and frozen in aliquots at −80°C.

### Determining the infectious titer of RVPs

Because the relationship between infected cells and virus dose is typically not linear for flaviviruses, the titers of all RVP stocks were measured by serial dilution. Serial two-fold dilutions of RVP-containing supernatants were used to infect Raji cells that express the attachment factor DC-SIGNR. Infection was measured 48 h post-infection by flow cytometry. Only data obtained from the linear portion of the resulting virus dose-response curves was used for analysis.

### Measurements of kinetic aspects of antibody-mediated neutralization and ADE

#### Single concentration studies

WNV RVP stocks were diluted in the presence or absence of 10 ng/ml MAb E16 or 200 ng/ml MAb 3H5 in a total volume of ∼7 ml and pre-incubated for one hour at room temperature to allow the binding reaction to reach equilibrium. The virus mixtures were further incubated at 37°C for up to four days, during which 450 µl samples were removed at indicated times and frozen for analysis. Two-fold dilutions of RVPs from each sample were used to infect Raji-DC-SIGNR cells at 37°C (200 µl total volume). Infectivity was determined 48 h post-infection by flow cytometry. Using values corresponding to the linear portion of the curve, infectivity was normalized to levels obtained prior to incubation at 37°C (but after the initial room temperature incubation) and fitted to a single-phase exponential decay to obtain the half-life (GraphPad Prism; GraphPad Software, La Jolla, CA).

#### Dose-response studies

To assess the kinetic aspects of neutralization over a range of antibody concentrations, WNV RVP stocks were diluted into the linear range of infectivity and incubated with serial dilutions of the indicated MAbs, Fab fragments, or polyclonal sera. To ensure our experiments were performed under conditions of antibody excess, antibody dose-response studies were performed using multiple dilutions of RVPs. These studies confirmed that the concentration of RVPs does not affect neutralization potency (EC_50_), as predicted by the assumptions of the law of mass action and the percentage law [65], [67]. Antibody-virus complexes were initially incubated for one hour at room temperature to allow the binding reaction to reach equilibrium, and then further incubated at 33°C, 37°C, or 40°C for incremental lengths of time, at which point the infectivity of virus-antibody complexes was determined by infecting Raji-DC-SIGNR target cells. Infections were carried out at 37°C (300 µl total volume) and infectivity was monitored by flow cytometry 48 h post-infection. In addition to the half-life data at each concentration of MAb or dilution of sera (calculated as described above), dose-response curves at each time point were analyzed by non-linear regression (variable slope) to predict the EC_50_ value using GraphPad Prism (GraphPad Software, La Jolla, CA). In a subset of experiments, K562 cells that express the activating human FcγIIb-receptor CD32a were used instead of Raji-DC-SIGNR cells in order to assess kinetic changes in ADE profiles. Neutralization studies with DENV-1 RVPs were performed analogous to WNV-Raji-DC-SIGNR infections, except the initial one hour incubation allowing for equilibrium was performed at 37°C instead of at room temperature.

### Quantitative real-time PCR

The genomic RNA content of RVP populations was measured using a modification of a previously described protocol [68]. Briefly, RVP containing supernatants were treated with 100 U recombinant DNase I, followed by RNA isolation using the QiaAmp Viral RNA Mini kit per the manufacturer's instructions (Qiagen, Valencia, CA). Amplification of viral genomic RNA was accomplished using the Superscript III One-Step RT-PCR system (Invitrogen, Carlsbad, CA) and primers specific for the 3′ untranslated region of the WNV lineage II replicon [69].

### Virus binding studies

Standard preparations of WNV RVPs were incubated with Raji-DC-SIGNR cells at 37°C in the absence or presence of the MAb E16 for incremental lengths of time to allow virus-cell binding to occur. Target cells were incubated in the presence of 20 mM NH_4_Cl to prevent virus fusion and genomic RNA replication. At the indicated times, cells were washed extensively with media and lysed. Total RNA was isolated using the QIAshredder and RNeasy Mini kits in accordance with the manufacturer's instructions (Qiagen, Valencia, CA). The relative amount of bound WNV RNA was enumerated by quantitative real-time PCR using a DNA molecular clone plasmid as a standard [49], [68]. The resulting kinetic data was fit to a one-phase association model using GraphPad Prism (GraphPad Software, La Jolla, CA).

## Supporting Information

Figure S1 **Virus-antibody steady-state binding.** Standard preparations of WNV or DENV-1 RVPs were incubated with serial four-fold dilutions of the indicated MAbs for the specified time periods prior to the addition of Raji-DC-SIGNR target cells. Infectivity was carried out at 37°C and monitored by flow cytometry at 48 h post-infection. Dose-response curves from representative experiments are expressed relative to the level of infectivity in the absence of antibody for each time point. (**A**) No significant difference in the EC_50_ of MAb E16 was observed whether cells were added immediately to RVP-antibody complexes (0 min) or after 15, 30, 45, or 60 minutes of incubation at room temperature (n = 3; p = .83). Similar experiments were performed with MAb E60, which binds the DII-fusion loop with high affinity, mediates neutralization primarily by blocking attachment to target cells [28], and is cross-reactive for DENV. No differences in the E60 neutralization dose-response profiles were observed whether cells were added to WNV (**B**) or DENV-1 (**C**) RVP-antibody complexes immediately or after 60 minutes of incubation at room temperature or 37°C. Additionally, similar results were obtained using the WNV-specific MAb E24 and WT WNV RVPs (**D**) or a variant incorporating a T330I mutation in the DIII-lateral ridge epitope recognized by this antibody (**E**). E24 binds with significantly reduced affinity to T330I WNV (too low to be measured by ELISA) and has been shown previously to poorly neutralize this variant [38]. No differences were observed whether cells were added to virus-antibody complexes immediately or after 30 or 60 minutes of incubation at room temperature. Overall, these results demonstrate the fast kinetics of antibody binding, and indicate that incubation for one hour (either at RT or 37°C) is sufficient for steady-state binding of antibody to WNV. Error bars display the standard error of duplicate infections. Data is representative of three (**A**), two (**B and C**), and one (**D and E**) independent experiment(s). RT = room temperature.
(TIF)

Figure S2 **The reduction in WNV infectivity in the presence of antibody cannot be explained by adherence of antibody-virus complexes to tissue culture plastic, the activation of complement, or the activity of proteases in the culture media.** (**A**) To rule out the impact of antibody-virus complexes adhering to tissue culture plastic and thereby disappearing from solution, we used quantitative RT-PCR to measure the amount of WNV RNA in samples collected immediately after the room temperature incubation required to achieve steady-state binding as compared to samples collected after lengthy incubation at 37°C (>72 h), either in the absence or presence of 10 ng/ml E16 (purple and blue bars, respectively). The data is presented as the ratio of WNV genomes present prior to incubation at 37°C versus after incubation. The error bars represent the standard error of five independent experiments (p = .32). (**B and C**) WNV RVPs were incubated in the absence or presence of 10 ng/ml E16 and, in some cases, a protease inhibitor (PI) cocktail (0.1×, Sigma-Aldrich) for one hour at room temperature to allow binding to reach equilibrium, after which the RVP-antibody complexes were incubated at 37°C. At incremental times, the infectivity of RVPs removed from 37°C incubation was determined following infection of Raji-DC-SIGNR cells. Infectivity was monitored by flow cytometry at 48 h post-infection and normalized to levels obtained prior to incubation at 37°C (but after equilibrium was reached). Normalized infectivity data was fitted to a single-phase exponential decay to obtain the half-life. The fold-decrease in RVP half-life was calculated by comparison to the half-life of RVPs incubated alone (the intrinsic decay rate). (**B**) To rule out a role for complement activation, we utilized an engineered E16 variant, chE16 N297Q, that cannot bind the complement component C1q [38], [42]. WNV RVPs were incubated in the presence of 10 ng/ml chE16 (control MAb with intact C1q binding ability) or 10 ng/ml of the chE16 N297Q MAb (purple and blue bars, respectively). The error bars represents the standard error of five independent experiments (p = .17). (**C**) To rule out the impact of contaminating proteases in solution, WNV RVPs were incubated in the presence of PI cocktail alone, 10 ng/ml E16 alone, or both (purple, blue, and green bars, respectively). The error bars represent the standard error of two independent experiments (p = .74, blue vs. green bars).
(TIF)

Figure S3 **The effects of epitope accessibility on kinetic increases in neutralization.** WNV neutralization is a multiple-hit phenomenon achieved when an individual virion is bound by antibody with a stoichiometry that exceeds a required threshold; our estimate of this threshold is 30 antibodies per virion (red dashed line) (reviewed by [29]). From this perspective, neutralization of flavivirus virions is governed by antibody affinity/avidity and epitope accessibility (the number of epitopes available for antibody binding). Most of the known flavivirus epitopes recognized by antibodies are poorly accessible on the mature virion due to steric constraints arising from the complex pseudo-icosahedral arrangement of E proteins [38], [39], [40], [52]. Changes in epitope accessibility that occur during virion maturation have been shown to significantly impact antibody-mediated neutralization of WNV [21], [52]. Epitope accessibility may also vary as a function of the dynamic motion, or “breathing”, of flavivirus E proteins on the mature virus particle. An increase in epitope accessibility via dynamic motion results in time-dependent increases in neutralization. As the number of accessible epitopes on the individual virion increases, the fraction of them that must be bound in order to exceed the stoichiometric threshold (percent occupancy) is reduced. For example, MAb E16 recognizes a relatively accessible epitope on the lateral ridge of DIII (shown as red spheres, adapted from [38]); 120 of 180 E proteins can be bound on the “average” state of the mature WNV virion [27], [28]. From this perspective, neutralization by E16 requires occupancy of 25% of the accessible epitopes on the virion. Increases in accessibility of the remaining 60 epitopes through dynamic motion of the E proteins result in a modest increase in neutralization potency; should all epitopes on the virion become accessible, neutralization will occur at an occupancy of 17% of the E proteins. By comparison, MAb E22 binds an epitope that is inaccessible on the mature virion (shown as red spheres, adapted from [38]). Most mature virions cannot be neutralized by E22 because they do not display a sufficient number of epitopes to allow for neutralization even when in the presence of saturating concentrations of antibody. Over time, dynamic exposure of the E22 epitope reduces the proportion of virions refractory to neutralization because the number of accessible epitopes on the virion becomes larger than the requirements of the neutralization threshold. Consequently, the occupancy requirements for neutralization are reduced, allowing neutralization of virions at lower concentrations of antibody. Because the number of epitopes available for binding by E16 on the mature virion is significantly greater than the cryptic determinants bound by E22, changes in neutralization occur more rapidly. In contrast, virions that display a very small number of epitopes relative to the stoichiometric threshold (such as for E22) may become sensitive to neutralization relatively slowly because the number of epitopes that must become accessible and s[ly bound by antibody is relatively large.
(TIF)

Figure S4 **Differences in the rate of kinetic changes in neutralization for WNV and DENV-1 using a cross-reactive MAb.** Nine serial four-fold dilutions of the cross-reactive DII-fusion loop-specific MAb E60 were incubated with WNV and DENV-1 RVPs for one hour at 37°C to allow binding to reach equilibrium. RVP-antibody complexes were then incubated at 37°C or 40°C for the indicated lengths of time before infecting Raji-DC-SIGNR cells. Infectivity was monitored by flow cytometry at 48 h post-infection. The reference curve represents RVP-antibody complexes added to Raji-DC-SIGNR cells immediately after the one hour incubation required to achieve steady-state binding. Dose-response curves are expressed relative to the infectivity of RVPs in the absence of antibody at each time point. Error bars display the standard error of duplicate infections. Results are representative of two independent experiments.
(TIF)

Figure S5 **Limits of the kinetic changes in neutralization and ADE.** Nine serial four-fold dilutions of MAb E16 were incubated with WNV RVPs for one hour at room temperature to allow binding to reach equilibrium. RVP-antibody complexes were then incubated at 37°C for incremental lengths of time before infecting Raji-DC-SIGNR (**A**) or K562 (**B**) cells. Infectivity was monitored by flow cytometry at 48 h post-infection. The reference curve represents RVP-antibody complexes added to cells immediately after the room temperature incubation. Dose-response curves from representative experiments are expressed relative to the infectivity in the absence of antibody at each individual time point (**A**) or, in the case of ADE, the maximum infectivity at each time point (**B**). Error bars display the standard error of duplicate infections. Data is representative of four (**A**) and three (**B**) independent experiments.
(TIF)

Figure S6 **WNV attachment to cells is relatively slow.** WNV RVPs were bound to Raji-DC-SIGNR cells for up to ∼400 minutes at 37°C in the absence (purple) or presence of sub-neutralizing (green) or neutralizing (blue) concentrations of the MAb E16. Virus binding was measured by tracking the presence of the viral genome using quantitative real-time PCR. Viruses were bound to cells for the indicated times and washed extensively before RNA analysis. Cells were treated with ammonium chloride to block viral fusion in the endosome and prevent the synthesis of viral RNA following infection. (**A**) The amount of WNV RNA bound is expressed as a function of the maximum binding in the absence of antibody. Error bars represent the standard error of duplicate measurements. (**B**) The resulting kinetic data was fit to a one-phase association model to obtain the binding rate constant. The means of five independent experiments are shown; error bars represent the standard errors.
(TIF)
