# Supplementary figures and images for: A Dynamic Landscape for Antibody Binding Modulates Antibody-Mediated Neutralization of West Nile Virus
Source: PLoS Pathog. 2011 Jun 30;7(6):e1002111. doi: 10.1371/journal.ppat.1002111 (PMC3128118; doi:10.1371/journal.ppat.1002111)

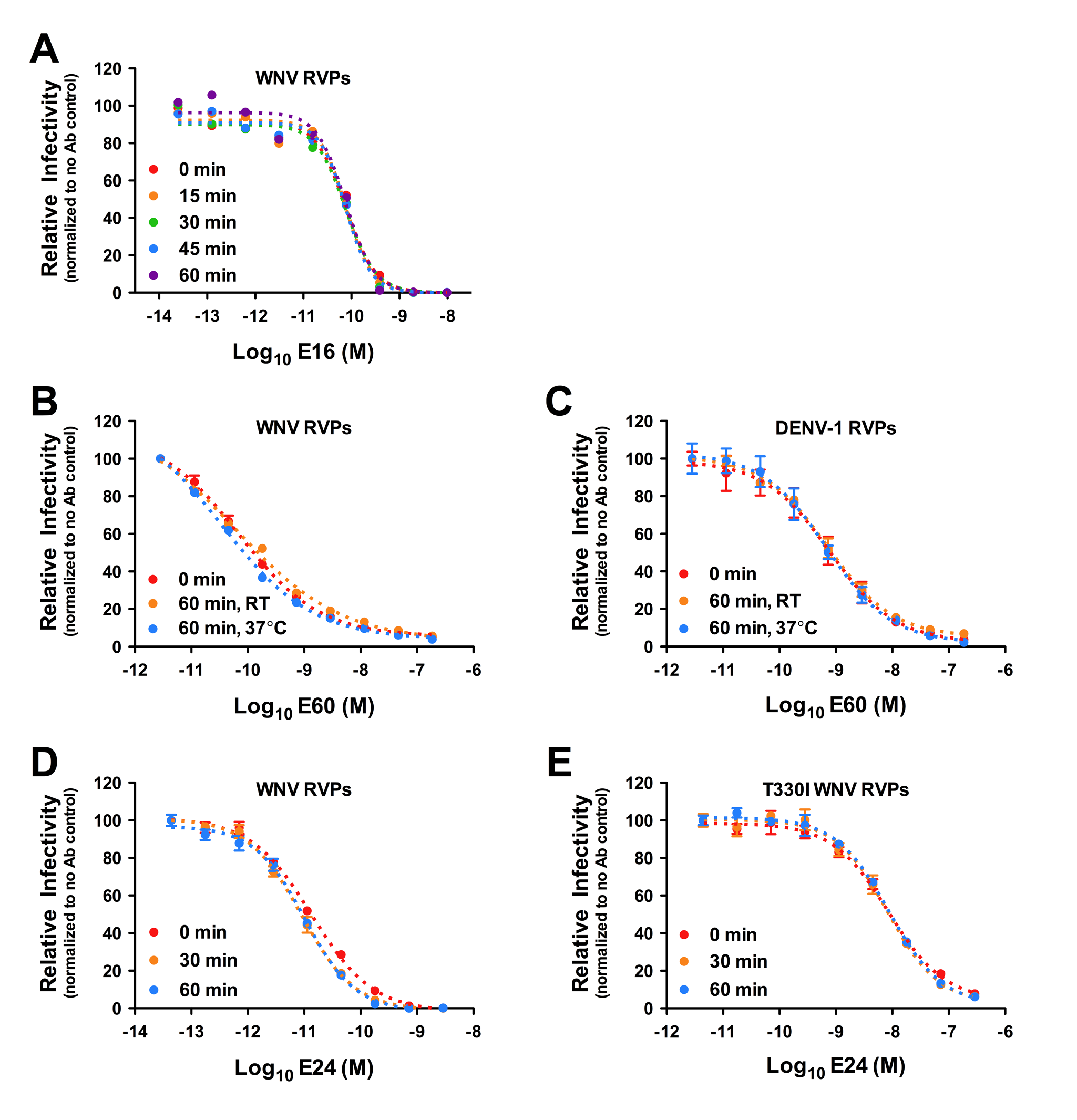

Supplement: Figure S1 — Virus-antibody steady-state binding. Standard preparations of WNV or DENV-1 RVPs were incubated with serial four-fold dilutions of the indicated MAbs for the specified time periods prior to the addition of Raji-DC-SIGNR target cells. Infectivity was carried out at 37°C and monitored by flow cytometry at 48 h post-infection. Dose-response curves from representative experiments are expressed relative to the level of infectivity in the absence of antibody for each time point. (A) No significant difference in the EC50 of MAb E16 was observed whether cells were added immediately to RVP-antibody complexes (0 min) or after 15, 30, 45, or 60 minutes of incubation at room temperature (n = 3; p = .83). Similar experiments were performed with MAb E60, which binds the DII-fusion loop with high affinity, mediates neutralization primarily by blocking attachment to target cells [28], and is cross-reactive for DENV. No differences in the E60 neutralization dose-response profiles were observed whether cells were added to WNV (B) or DENV-1 (C) RVP-antibody complexes immediately or after 60 minutes of incubation at room temperature or 37°C. Additionally, similar results were obtained using the WNV-specific MAb E24 and WT WNV RVPs (D) or a variant incorporating a T330I mutation in the DIII-lateral ridge epitope recognized by this antibody (E). E24 binds with significantly reduced affinity to T330I WNV (too low to be measured by ELISA) and has been shown previously to poorly neutralize this variant [38]. No differences were observed whether cells were added to virus-antibody complexes immediately or after 30 or 60 minutes of incubation at room temperature. Overall, these results demonstrate the fast kinetics of antibody binding, and indicate that incubation for one hour (either at RT or 37°C) is sufficient for steady-state binding of antibody to WNV. Error bars display the standard error of duplicate infections. Data is representative of three (A), two (B and C), and one (D and E) indep [file ppat.1002111.s001.tif]

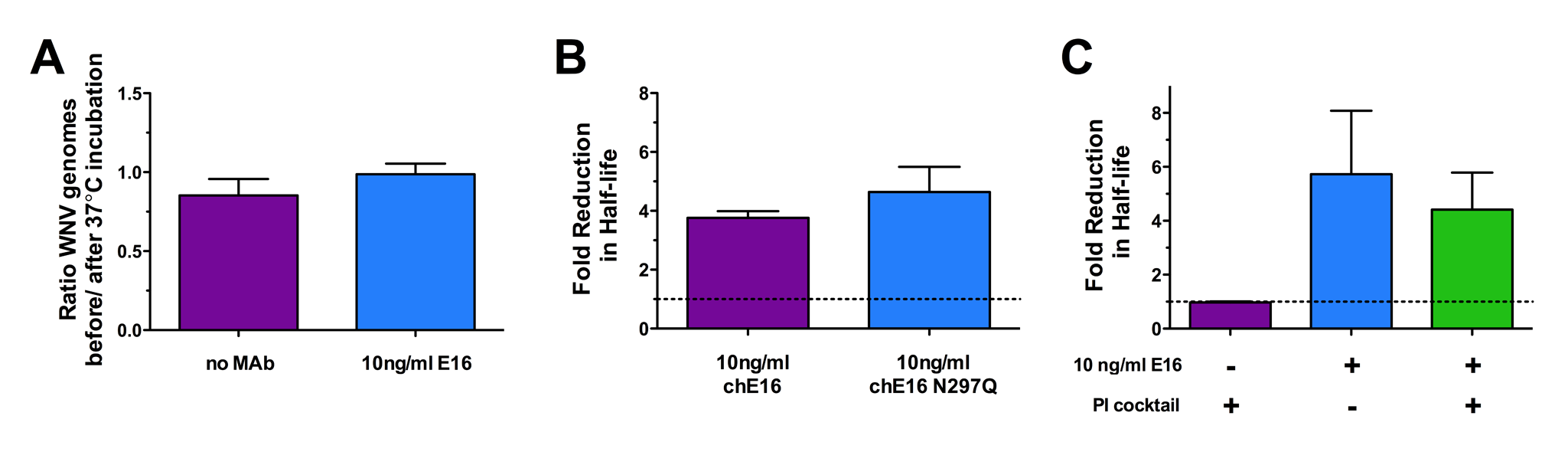

Supplement: Figure S2 — The reduction in WNV infectivity in the presence of antibody cannot be explained by adherence of antibody-virus complexes to tissue culture plastic, the activation of complement, or the activity of proteases in the culture media. (A) To rule out the impact of antibody-virus complexes adhering to tissue culture plastic and thereby disappearing from solution, we used quantitative RT-PCR to measure the amount of WNV RNA in samples collected immediately after the room temperature incubation required to achieve steady-state binding as compared to samples collected after lengthy incubation at 37°C (>72 h), either in the absence or presence of 10 ng/ml E16 (purple and blue bars, respectively). The data is presented as the ratio of WNV genomes present prior to incubation at 37°C versus after incubation. The error bars represent the standard error of five independent experiments (p = .32). (B and C) WNV RVPs were incubated in the absence or presence of 10 ng/ml E16 and, in some cases, a protease inhibitor (PI) cocktail (0.1×, Sigma-Aldrich) for one hour at room temperature to allow binding to reach equilibrium, after which the RVP-antibody complexes were incubated at 37°C. At incremental times, the infectivity of RVPs removed from 37°C incubation was determined following infection of Raji-DC-SIGNR cells. Infectivity was monitored by flow cytometry at 48 h post-infection and normalized to levels obtained prior to incubation at 37°C (but after equilibrium was reached). Normalized infectivity data was fitted to a single-phase exponential decay to obtain the half-life. The fold-decrease in RVP half-life was calculated by comparison to the half-life of RVPs incubated alone (the intrinsic decay rate). (B) To rule out a role for complement activation, we utilized an engineered E16 variant, chE16 N297Q, that cannot bind the complement component C1q [38], [42]. WNV RVPs were incubated in the presence of 10 ng/ml chE16 (control MAb with intact C1q binding ability) or 10 ng/ml of the c [file ppat.1002111.s002.tif]

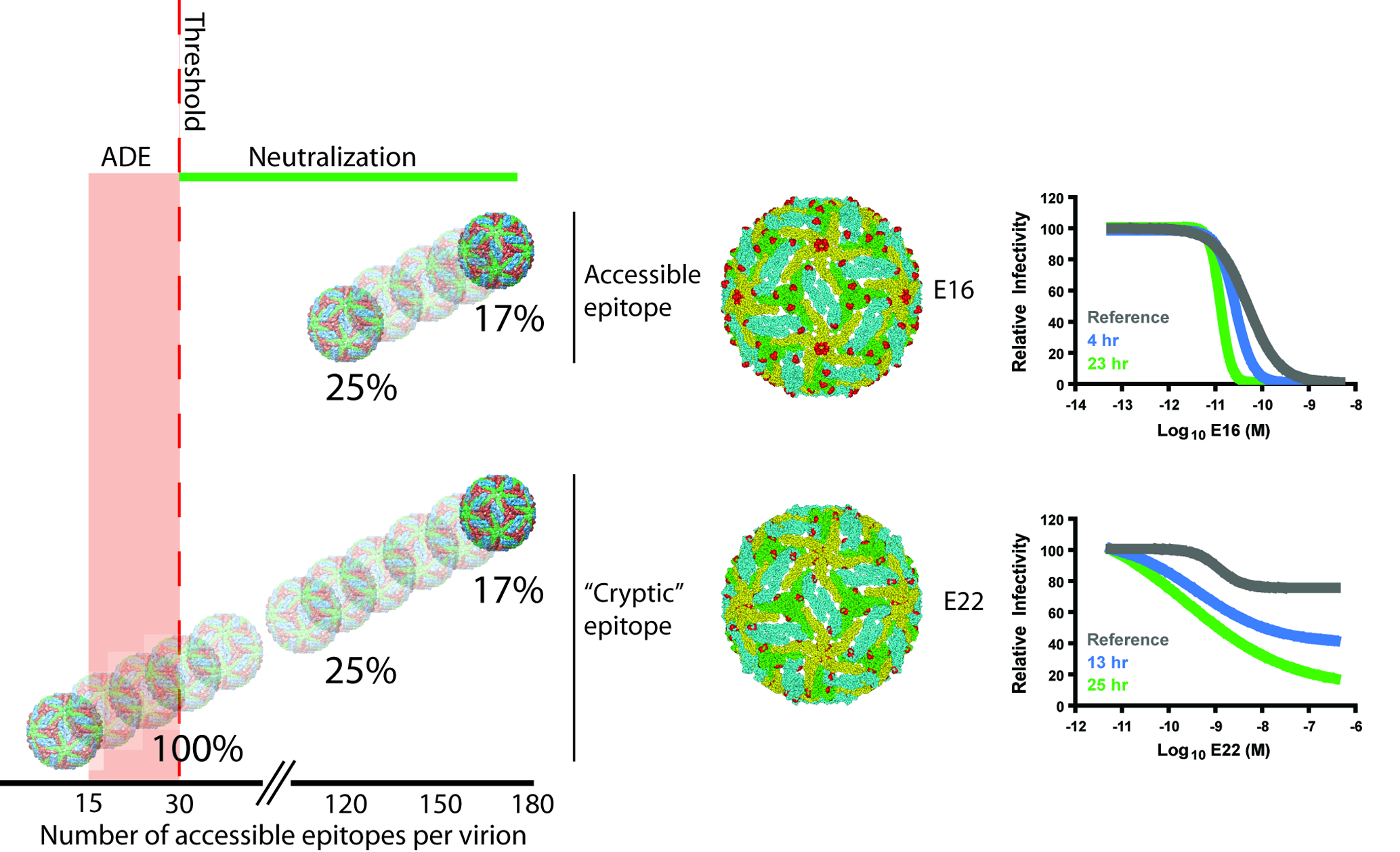

Supplement: Figure S3 — The effects of epitope accessibility on kinetic increases in neutralization. WNV neutralization is a multiple-hit phenomenon achieved when an individual virion is bound by antibody with a stoichiometry that exceeds a required threshold; our estimate of this threshold is 30 antibodies per virion (red dashed line) (reviewed by [29]). From this perspective, neutralization of flavivirus virions is governed by antibody affinity/avidity and epitope accessibility (the number of epitopes available for antibody binding). Most of the known flavivirus epitopes recognized by antibodies are poorly accessible on the mature virion due to steric constraints arising from the complex pseudo-icosahedral arrangement of E proteins [38], [39], [40], [52]. Changes in epitope accessibility that occur during virion maturation have been shown to significantly impact antibody-mediated neutralization of WNV [21], [52]. Epitope accessibility may also vary as a function of the dynamic motion, or “breathing”, of flavivirus E proteins on the mature virus particle. An increase in epitope accessibility via dynamic motion results in time-dependent increases in neutralization. As the number of accessible epitopes on the individual virion increases, the fraction of them that must be bound in order to exceed the stoichiometric threshold (percent occupancy) is reduced. For example, MAb E16 recognizes a relatively accessible epitope on the lateral ridge of DIII (shown as red spheres, adapted from [38]); 120 of 180 E proteins can be bound on the “average” state of the mature WNV virion [27], [28]. From this perspective, neutralization by E16 requires occupancy of 25% of the accessible epitopes on the virion. Increases in accessibility of the remaining 60 epitopes through dynamic motion of the E proteins result in a modest increase in neutralization potency; should all epitopes on the virion become accessible, neutralization will occur at an occupancy of 17% of the E proteins. By comparison, MAb E22 binds a [file ppat.1002111.s003.tif]

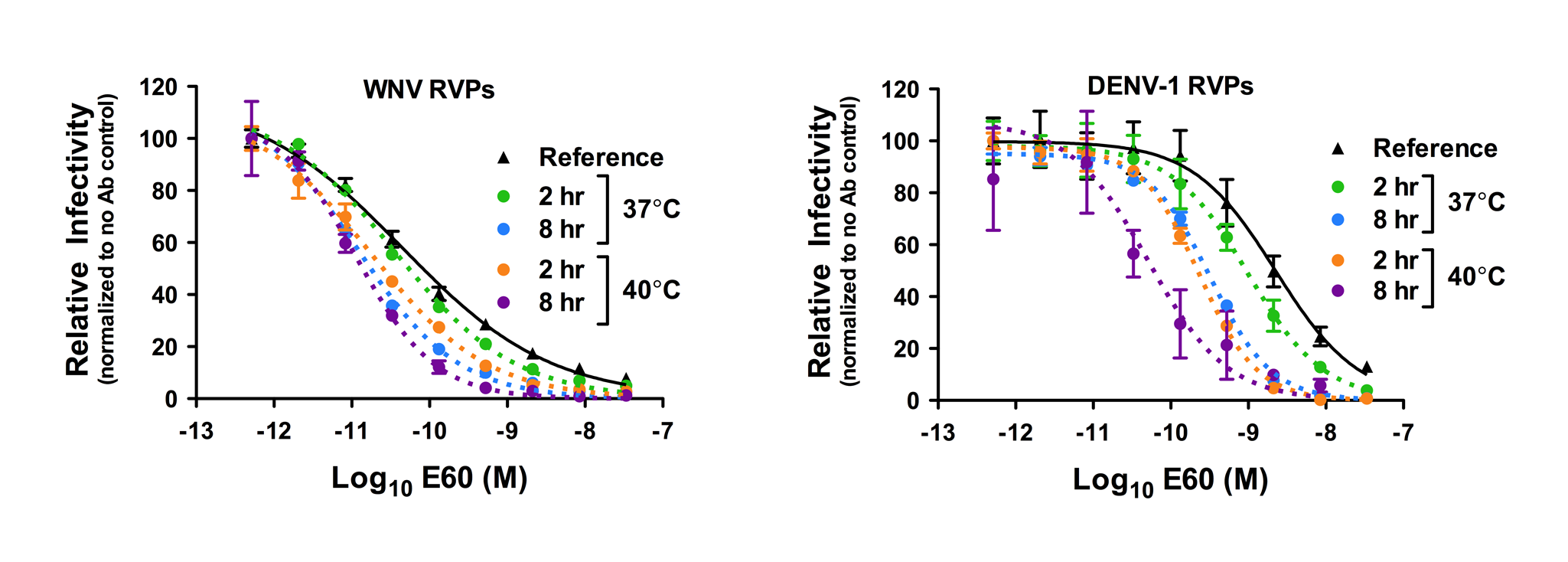

Supplement: Figure S4 — Differences in the rate of kinetic changes in neutralization for WNV and DENV-1 using a cross-reactive MAb. Nine serial four-fold dilutions of the cross-reactive DII-fusion loop-specific MAb E60 were incubated with WNV and DENV-1 RVPs for one hour at 37°C to allow binding to reach equilibrium. RVP-antibody complexes were then incubated at 37°C or 40°C for the indicated lengths of time before infecting Raji-DC-SIGNR cells. Infectivity was monitored by flow cytometry at 48 h post-infection. The reference curve represents RVP-antibody complexes added to Raji-DC-SIGNR cells immediately after the one hour incubation required to achieve steady-state binding. Dose-response curves are expressed relative to the infectivity of RVPs in the absence of antibody at each time point. Error bars display the standard error of duplicate infections. Results are representative of two independent experiments. (TIF) [file ppat.1002111.s004.tif]

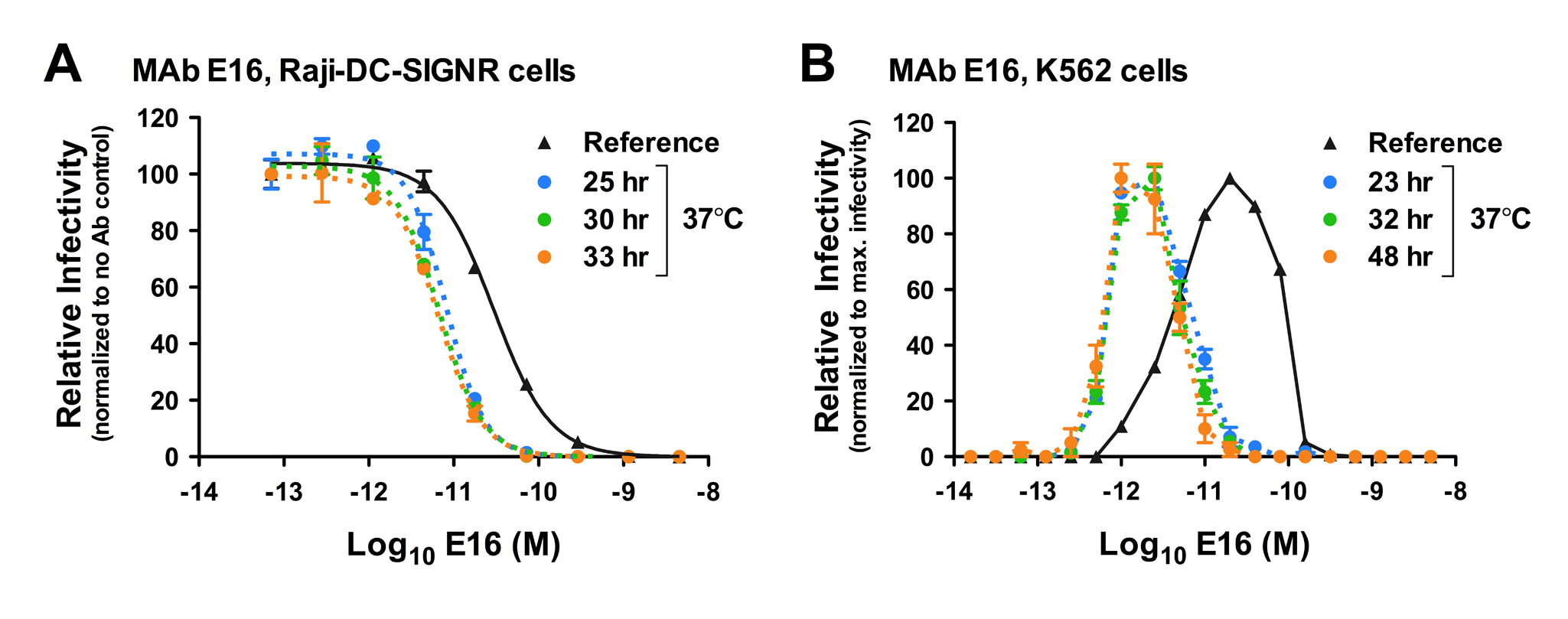

Supplement: Figure S5 — Limits of the kinetic changes in neutralization and ADE. Nine serial four-fold dilutions of MAb E16 were incubated with WNV RVPs for one hour at room temperature to allow binding to reach equilibrium. RVP-antibody complexes were then incubated at 37°C for incremental lengths of time before infecting Raji-DC-SIGNR (A) or K562 (B) cells. Infectivity was monitored by flow cytometry at 48 h post-infection. The reference curve represents RVP-antibody complexes added to cells immediately after the room temperature incubation. Dose-response curves from representative experiments are expressed relative to the infectivity in the absence of antibody at each individual time point (A) or, in the case of ADE, the maximum infectivity at each time point (B). Error bars display the standard error of duplicate infections. Data is representative of four (A) and three (B) independent experiments. (TIF) [file ppat.1002111.s005.tif]

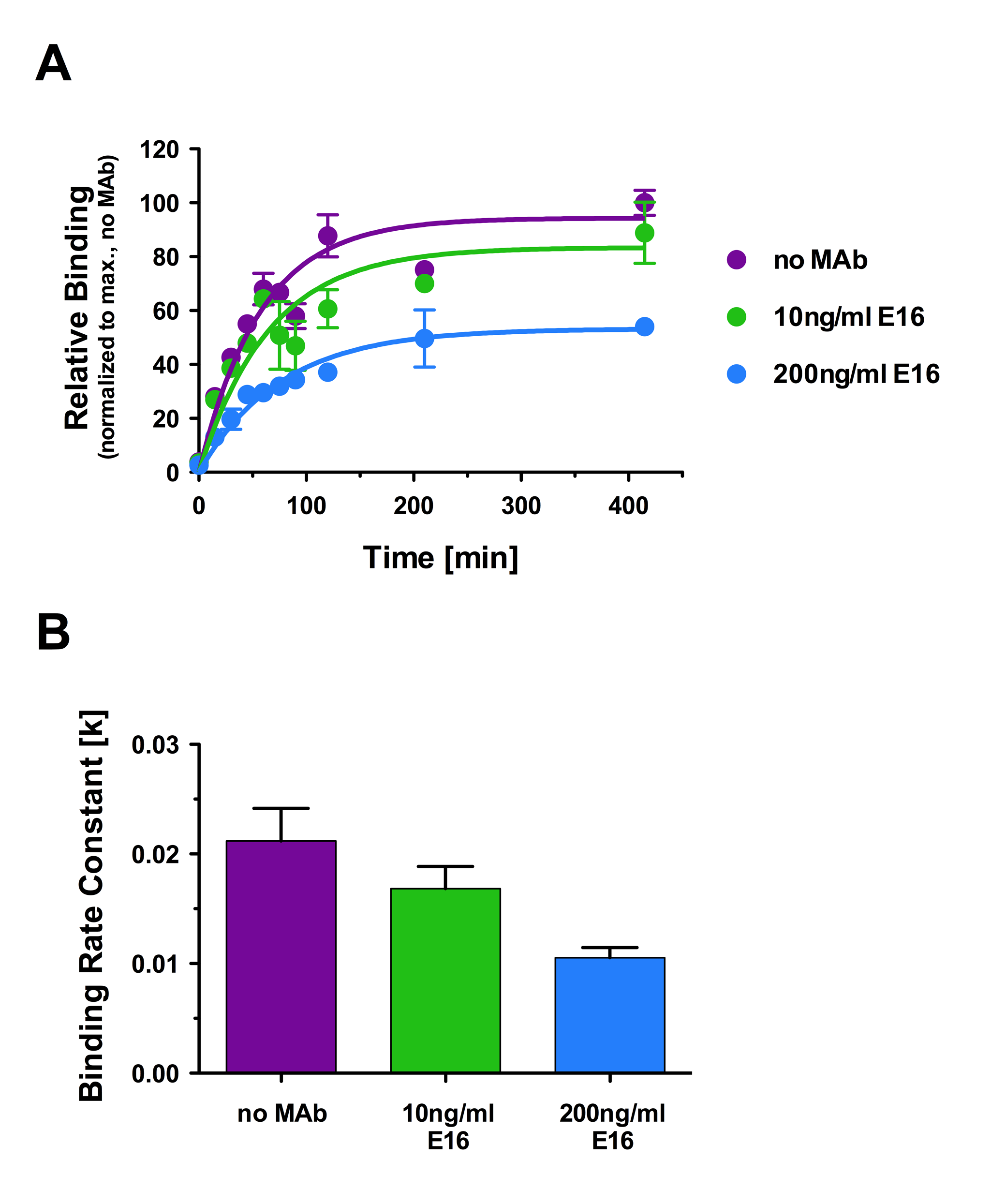

Supplement: Figure S6 — WNV attachment to cells is relatively slow. WNV RVPs were bound to Raji-DC-SIGNR cells for up to ∼400 minutes at 37°C in the absence (purple) or presence of sub-neutralizing (green) or neutralizing (blue) concentrations of the MAb E16. Virus binding was measured by tracking the presence of the viral genome using quantitative real-time PCR. Viruses were bound to cells for the indicated times and washed extensively before RNA analysis. Cells were treated with ammonium chloride to block viral fusion in the endosome and prevent the synthesis of viral RNA following infection. (A) The amount of WNV RNA bound is expressed as a function of the maximum binding in the absence of antibody. Error bars represent the standard error of duplicate measurements. (B) The resulting kinetic data was fit to a one-phase association model to obtain the binding rate constant. The means of five independent experiments are shown; error bars represent the standard errors. (TIF) [file ppat.1002111.s006.tif]
